# Association between height and malignancy among children in the north of Iran

**Published:** 2015-04-20

**Authors:** B Darbandi, A Baghersalimi, M Jafroodi, Z Atrkarroshan, SH Koohmanaei, A Hassanzadeh rad, S Dalili

**Affiliations:** 1 Pediatric Growth Disorders Research Center, 17 Shahrivar Hospital, Guilan University of Medical Sciences, Rasht, Iran.; 2 Pediatric Endocrinologist, Guilan University of Medical Sciences, Rasht, Iran.; 3 Pediatric Hematologist/Oncologist, Guilan University of Medical Sciences, Rasht, Iran.

**Keywords:** Height, Malignancy, NCHS, Pediatric, Percentile

## Abstract

**Background:**

This study aim to determine the association between height and cancer in the children aged 14 years at the time of diagnosis in Rasht, Iran.

**Materials and Methods:**

In this cross-sectional study, height of patients with a malignancy (≤14) at the time of diagnosis measured in the standard charts of United States National Center for the Health Statistics (NCHS). Data were reported by descriptive statistics and analyzed by Regression tests in SPSS version 19.

**Results:**

Overall, 78 male (38.6%) and 124 female (61.4%) patients with various kinds of malignancies were evaluated for their heights. Leukemia was the most common type of cancer. The median height of the patients was more than 20^th^ percentile and under 50^th^ percentile of the NCHS. No significant association was found between height and leukemia.

**Conclusion:**

Previously, the median height of Iranian girls and boys (≤15) reported under 20^th^ percentile of the NCHS. In this study, the median height of the patients at the time of diagnosis was more than 20^th^ percentile of the NCHS. There was a correlation between height and cancer among our patients, although, this correlation can be assessed by further cohort study.

## Introduction

Height alteration is an anthropometric index which could induce and affect different diseases such as heart disease and respiratory disorders (1).

According to previous prospective investigations in adults, there is significant association between height and risk of cancer (1). Recent studies demonstrated a significant relation between raised body mass index (BMI) and height with Hodgkin Lymphoma (HL) (2,3). In addition, increased incidence of the osteosarcoma was seen in taller individuals and those with earlier pubertal growth spurts (2, 3). It is hypothesized that this may be correlated with growth-related genes which were associated with vitamin D receptor polymorphisms (4). Furthermore, height was indicated as a main feature for increased rate of shared site cancers in males. It was mentioned that 

height related issues such as rate of susceptible cells and childhood growth-influencing exposures lead to this increased rate (5). The most reliable relations were noted for adulthood breast cancer in adults (6). In addition to adulthood assessments, there were few studies which investigated association between height and childhood malignancies. They presented controversial results in which at primordial diagnosis, ALL in children and adults was noted in taller patients (7, 8). However, some investigators didn’t find significant relation between elevated height and childhood malignancy. They showed that patients with solid tumors had lower weight (for height) index versus leukemic patients (9). According to the few childhood studies and controversial results, we aimed to investigate association between height and malignancy in the patients referred to 17 Shahrivar pediatrics hospital in north of Iran.

## Materials and Methods

This was an analytic cross-sectional study which included newly diagnosed children aged 14 years or less with a malignancy who admitted to pediatric oncology ward in 17 Shahrivar Children Hospital between 2000 and 2013 in Rasht, Iran. For the children less than 2 years of age recumbent height was assessed. Patients laid down on their back on a firm table and one person hold the head against the headboard with both hands. The second person gently flattened the knees and flexed the ankles of the patients to 90 degrees and brought the footboard up to the flat soles of the flexed feet. The length measurement was then read off the scale to the nearest 1/2 cm. For the patients aged over 2 years, height was measured in standing upright position with bare feet, while closed heels, buttocks, shoulders and occiput touched the stadiometer. Their heights were measured up to 200 centimeters. According to the lack of growth charts which specifically created and standardized for Iranian children and adolescents, the authors applied US NCHS charts for growth monitoring and assessed height status in pediatric population (12). 

According to the previous studies, the median height and weight of the Iranian healthy children aged less than 15 years were under 20^th^ percentile of the NCHS (13,14). 

They recommended that for boys and girls up to 14 years, the comparison must be evaluated based on 25^th^, 50^th^ percentile heights (13, 14). Therefore, in this study, investigators applied their recommendation. 

**Statistical Analysis**

In this study, data were reported by descriptive statistics and analyzed by Regression tests in SPSS version 19.

## Results

Two hundred and two patients including 78(38.6%) boys and 124(61.4%) girls were evaluated. Results showed that leukemia was the most common cause of malignancy .The frequencies of malignancies were summarized in Table 1. 

Mean heights in children with malignancies were more than 25^th^ percentile, but less than 50^th^ percentile of the US NCHS charts, except for lymphoma, fibrosarcoma and retinoblastoma which were even less than 25^th^. However, there was no significant difference between mean heights of children with malignancies in comparisons with 25^th^ and 50^th^ percentile of US NCHS charts except for patients with Hodgkin lymphoma. The comparison of heights with normal values of 25^th^and 50^th^ percentile heights, based on age had been summarized in Tables 2 and 3. Mean heights of children with malignancies according to their sex in comparison with 25^th^ and 50^th^percentiles of US NCHS charts had been noted respectively in Table 4and 5.Comparison of height median of children with malignancies with 25^th^ and 50^th^ percentile of US NCHS chart had been summarized in Table 6.

**Table 1:** Frequency of different malignancies among newly diagnosed children

Variable		Frequency	%	Total
Leukemia	ALL	102	50.5	62.4
	AML	24	11.9	
Lymphoma	HL	7	3.5	11.4
	NHL	16	7.9	
Brain tumor	Brain tumor	12	6.0	6.0
Neuroblastoma	Neuroblastoma	11	5.4	5.4
RMS	RMS	9	4.5	4.5
Wilms’ Tumor	Wilms’ Tumor	4	2.0	2.0
Ewing	Ewing	8	4.0	4.0
Fibrosarcoma	Fibrosarcoma	3	1.5	1.5
Retinoblastoma	Retinoblastoma	3	1.5	1.5
Germ cell tumor	Germ cell tumor	2	1.0	1.0
Hepatoblastoma	Hepatoblastoma	1	0.5	0.5
Total		202	100	100

**Table 2:** Comparison of the patient’s heights with50^th^ percentile of US NCHS chart

Variable		Mean of Height	N	Mean of SD score	Mean of SE score	p-value
Leukemia	ALL	112.9450	100	21.42168	2.14217	0.239
	NCHSS50	113.9690	100	20.69071	2.06907	
	AML	111.2292	24	26.77360	5.46514	0.274
	NCHS 50	113.3167	24	26.34722	5.37810	
Neuroblastoma	Neuroblastoma	105.9000	10	24.65067	7.79523	0.520
	NCHSS50	104.8200	10	25.01199	7.90949	
Lymphoma	HL	119.5714	7	9.51940	3.59800	0.013
	NCHSS50	129.1286	7	11.90640	4.50020	
	NHL	131.3750	16	18.86399	4.71600	0.633
	NCHSS50	130.4437	16	20.87400	5.21850	
RMS	RMS	118.0556	9	34.82316	11.60772	0.644
	NCHsS50	119.3444	9	35.62657	11.87552	
Wilms’ Tumor	Wilms’ Tumor	124.5000	4	8.96289	4.48144	0.566
	NCHSS50	126.6750	4	14.35627	7.17814	
Brain tumor	Brain tumor	111.4545	11	23.52600	7.09336	0.271
	NCHS 50	113.5545	11	22.33797	6.73515	
EWING	Ewing	123.0000	8	23.76672	8.40281	0.215
	NCHS 50	125.7625	8	21.14203	7.47484	
Fibrosarcoma	Fibrosarcoma	105.6667	3	37.42103	21.60504	0.429
	NCHSS50	118.0333	3	44.85558	25.89738	
Retinoblastoma	Retinoblastoma	85.6667	3	9.60902	5.54777	0.410
	NCHSS50	98.6333	3	12.22675	7.05912	
Germ cell tumor	Germ cell tumor	113.5000	2	33.23402	23.50000	0.493
	NCHSS50	94.3000	2	6.64680	4.70000	

**Table 3:** Comparison of the patient’s heights with 25^th^ percentile of US NCHS chart

Variable		Mean of Height	N	Mean of SD score	Mean of SE score	p-value
Leukemia	ALL	112.9450	100	21.42168	2.14217	0.01
	NCHSS25	110.6830	100	19.83860	1.98386	
	AML	111.2292	24	26.77360	5.46514	0.541
	NCHS 25	110.0833	24	25.25275	5.15470	
Neuroblastoma	Neuroblastoma	105.9000	10	24.65067	7.79523	0.034
	NCHSS25	101.9200	10	24.08083	7.61503	
Lymphoma	HL	119.5714	7	9.51940	3.59800	0.074
	NCHSS 25	125.2429	7	11.46761	4.33435	
	NHL	131.3750	16	18.86399	4.71600	0.018
	NCHSS 25	126.5000	16	20.07821	5.01955	
RMS	RMS	118.0556	9	34.82316	11.60772	0.415
	NCHSS 25	115.8000	9	34.27751	11.42584	
Wilms’ Tumor	Wilms’ Tumor	124.5000	4	8.96289	4.48144	0.646
	NCHSS 25	122.9000	4	13.80652	6.90326	
Brain tumor	Brain tumor	112.5	11	24.47	7.09336	0.541
	NCHSS 25	113.5545	11	22.33797	6.73515	
Ewing	Ewing	123.0000	8	23.76672	8.40281	0.661
	NCHSS 25	122.0250	8	20.30241	7.17799	
Fibrosarcoma	Fibrosarcoma	105.6667	3	37.42103	21.60504	0.532
	NCHSS25	114.6667	3	43.19842	24.94062	
Retinoblastoma	Retinoblastoma	85.6667	3	9.60902	5.54777	0.484
	NCHSS25	96.0667	3	11.68004	6.74347	
Germ cell tumor	Germ cell tumor	113.5000	2	33.23402	23.50000	0.459
	NCHS	91.8500	2	6.29325	4.45000	

**Table 4:** Comparison between mean and standard deviation of 25^th^ percentile height in malignancies based on sex

Sex	Mean of Height(CM)	N	Mean of SD score	Mean of SE score	p-value
**Female**	112.9671	76	24.21602	2.77777	0.122
**NCHS 25**	111.3263	76	23.81339	2.73158	
**Male**	114.9098	122	23.06987	2.08865	0.02
**NCHS** **25**	112.9664	122	21.30008	1.92842	
**Total**	114.1641	198	23.47441	1.66825	0.005
**NCHS**	112.3369	198	22.25301	1.58145	

**Table 5:** Comparison between mean and standard deviation of 50^th^ percentile height in malignancies based on sex

Sex	Mean of Height(CM)	N	Mean of SD score	Mean of SE score	p-value
**Female**	112.9671	76	24.21602	2.77777	0.119
**NCHS** **S** **50**	114.6566	76	24.74739	2.83872	
**Male**	114.9098	122	23.06987	2.08865	0.09
**NCHS** **S** **50**	116.3246	122	22.23636	2.01319	
**Total**	114.1641	198	23.47441	1.66825	0.022
**NCHS** **S** **50**	115.6843	198	23.18453	1.64765	

**Table 6:** Comparison of height median of children with malignancies with 25^th^ and 50^th^ percentile of US NCHS chart.

**Variable**		**Median of** **Height**	**SD score**
**Leukemia**	ALL	110.0000	21.31518
	NCHS25	109.2000	19.94018
	NCHS50	112.4000	20.79901
	AML	100.0000	26.77360
	NCHS25	105.2500	25.25275
	NCHS 50	108.3500	26.34722
**Neuroblastoma**	Neuroblastoma	100.5000	24.65067
	NCHS25	98.9500	24.08083
	NCHS50	101.8000	25.01199
**Lymphoma**	HL	115.0000	9.51940
	NCHS25	127.3000	11.46761
	NCHS50	131.3000	11.90640
	NHL	133.0000	17.88641
	NCHS25	133.1000	19.27019
	NCHS50	137.3000	20.01986
**RMS**	RMS	129.000	34.82316
	NCHS25	127.4000	34.27751
	NCHS50	131.5000	35.62657
**Wilms’ Tumor**	Wilms’ Tumor	121.5000	8.96289
	NCHS25	116.3000	13.80652
	NCHS50	119.8000	14.35627
**Brain tumor**	Brain tumor	112.5000	24.47311
	NCHS25	104.7000	22.59480
	NCHSS50	107.8000	23.55402
**EWING**	EWING	122. 000	23.76
	NCHS 25	124.7000	20.30241
	NCHS50	128.6000	21.14203
**Fibrosarcoma**	Fibrosarcoma	92.0000	37.42103
	NCHS25	123.6000	43.19842
	NCHS50	127.5000	44.85558
**Retinoblastoma**	Retinoblastoma	84.0000	9.60902
	NCHS25	95.1000	11.68004
	NCHS50	97.7000	12.22675
**Germ cell tumor**	Germ cell tumor	113.5000	33.23402
	NCHS25	91.8500	6.29325
	NCHS50	94.3000	6.64680

## Discussion

Childhood cancer occurred due to the aberrations in early developmental process. The genetic processes which can lead to childhood cancer are likely different from adulthood. At least, the carcinogenic process in children has so much shorter time (2, 3, 10). Some of the pediatric malignancies are clearly related to genetic aberrations involving insulin like growth factors such as Ewing sarcoma, Rhabdomyosarcoma, and osteosarcoma (11, 12).

On the other hand, insulin like growth factors involve in many aspects of normal physiology (13, 14), which among them, growth is the most investigated (15). Height as an important parameter of growth is a product of many factors such as genetic, environmental, and nutritional factors. Previous studies have found a significant reduction in height of children with acute lymphoblastic leukemia (16, 17). But, according to our results, there was no significant relation between leukemia and height.

Based on previous studies, the median height and weight of the Iranian girls and boys aged less than 15 years were under 20^th^ percentile of the US NCHS charts (18, 19). In order to reduce this gap, cultural education along with the economic development is needed. (18, 19). Furthermore, Mohammad et al mentioned that median heights and weights of Iranian children up to 15 years of age were both below the 20th percentiles of NCHS standards; however, median heights and weights of 15-18 years participants laid on the 20^th^ and 25^th^ percentiles of NCHS, respectively. These results may suggested that the gap could be filled by nutritional and health services improvements along with the socio-economic developments (19). With the best of our knowledge, this is the first study about the correlation between height and pediatric malignancy in Iran. In this study we evaluated the height of 202 patients with different type of pediatric malignancies. We found that mean heights of children with malignancies were less than 50^th^ percentile NCHS but without significant difference (p>0.05) except for Hodgkin lymphoma. On the other hand, mean heights of this patients were significantly (p<0.05) more than 25^th^ percentile NCHS. In a study by Abtahi M and Mohammad K et al. they used median values of height to comapre Iranian children with NCHS charts (15). With consideration that in a normally distributed population median and mean values are the same, they found that 50^th^ percentile of healthy Iranian children less than 15 years of age laid on 20th percentiles of NCHS charts; so we can conclude that the mean height of our patients were significantly more than 50 percentile of the Iranian children.

There are few studies performed worldwide that evaluated correlation between height and pediatric malignancies. Our findings were inconsistent with Pui ***et al. who found no ***significant deviation from population norms in any of the 10 malignancies’ categories after proper adjustment for multiple significance testing(20). But our findings ***were consistent with*** Fraumeni JF ***et al*** who ***mentioned that*** tall stature and an earlier pubertal growth spurt might be noted as important factors in the etiology of both osteosarcoma and Ewing sarcoma (21). Furthermore, IGF-1 is a responsible factor for enhancing tumor development in certain types of human cancer and non-malignant diseases like benign prostatic hyperplasia(22). 

It can increase cancer risk, cell proliferation and suppression of apoptosis (23). Furthermore, Insulin-like growth factor 1 (IGF1) motivates mitosis and hinders apoptosis and can be modified by IGF binding protein 3 (IGFBP)(24). Therefore, decreased IGF-1 can indicate new strategies for cancer prevention. On the other hand ,the IGFBP-3/IGFBP-3R axis may present therapeutic and prognostic values for cancer therapy (25, 26). Therefore, it seems that assessing these factors could be beneficial. Our study has three major limitations. The relatively small sample size and unexpected cancer type distribution of our patients can be noted as two major limitations. As it was mentioned, brain tumors are in the second place of the most frequent pediatric malignancies, worldwide (about 20% of total) but it consist only six percent of our patients. In addition, the third limitation comes from the absence of a standardized nationwide growth chart for the Iranian children. Since this study is a clue, further, larger, and preferentially multicenter studies are mandatory which assess control group from the same geographical zone and with matched groups for age and sex. 

## Conclusion

In this study, the median height of the patients at the time of diagnosis was more than 20^th^ percentile of the NCHS. There was a correlation between height and cancer among our patients, although, this correlation can be assessed by further cohort study. 

## Conflict of interest

All authors declare that they have no conflict of interest
